# Ordinal Decision-Tree-Based Ensemble Approaches: The Case of Controlling the Daily Local Growth Rate of the COVID-19 Epidemic

**DOI:** 10.3390/e22080871

**Published:** 2020-08-07

**Authors:** Gonen Singer, Matan Marudi

**Affiliations:** 1 Faculty of Engineering, Bar-Ilan University, Ramat-Gan 52900, Israel; 2 Department of Industrial Engineering, Tel-Aviv University, Tel Aviv-Yafo 39040, Israel; mr.marudi@gmail.com

**Keywords:** decision trees, ensemble algorithms, random forest, AdaBoost, objective-based entropy, information gain, ordinal classification, COVID-19, epidemic

## Abstract

In this research, we develop ordinal decision-tree-based ensemble approaches in which an objective-based information gain measure is used to select the classifying attributes. We demonstrate the applicability of the approaches using AdaBoost and random forest algorithms for the task of classifying the regional daily growth factor of the spread of an epidemic based on a variety of explanatory factors. In such an application, some of the potential classification errors could have critical consequences. The classification tool will enable the spread of the epidemic to be tracked and controlled by yielding insights regarding the relationship between local containment measures and the daily growth factor. In order to benefit maximally from a variety of ordinal and non-ordinal algorithms, we also propose an ensemble majority voting approach to combine different algorithms into one model, thereby leveraging the strengths of each algorithm. We perform experiments in which the task is to classify the daily COVID-19 growth rate factor based on environmental factors and containment measures for 19 regions of Italy. We demonstrate that the ordinal algorithms outperform their non-ordinal counterparts with improvements in the range of 6–25% for a variety of common performance indices. The majority voting approach that combines ordinal and non-ordinal models yields a further improvement of between 3% and 10%.

## 1. Introduction

### 1.1. Conventional Approaches for Predicting the Dynamic Spread of an Epidemic

In epidemiology, mathematical modeling is widely used to predict the transmissibility and dynamic spread of an epidemic, while statistical analysis is often used to evaluate the effect of a variety of variables on epidemic transmission. For the prediction task, the most commonly used mathematical models are those that apply SIR/SEIR (susceptible, (exposed), infectious, and removed) differential equations [1,2,3,4]. These studies usually assume available data on the number of susceptible individuals and the numbers of infections, deaths, and recoveries. Recently, several studies have incorporated spatial patterns into epidemiological mathematical models for predicting the spread of an epidemic, by invoking specific assumptions regarding the behavior and location of individuals in the network [5,6,7]. Research studies that examine the effect of different factors on the spread of an epidemic tend to use statistical analysis techniques such as Pearson’s correlation coefficient, descriptive statistics, and regression models. Mecenas et al. [8], for example, described 17 recent studies that used these techniques to investigate the effect of weather variables on the spread of COVID-19 and SARS.

In comparison to that for weather variables, the evidence about the effect of environmental factors on the transmission and viability of COVID-19 and other epidemics is less conclusive. Pedersen and Meneghini [9], for example, explored the effects of containment measures and found that drastic restrictions have reduced the spread of COVID-19 modestly but have been insufficient to halt the epidemic. Mastrandrea and Barrat [10] showed that social interactions shape the patterns of epidemic spreading processes in populations and explored how incomplete data on contact networks affect the prediction of epidemic risk.

All of the aforementioned mathematical modeling approaches for spread prediction tend to be predicated on model-specific assumptions and, in general, cannot represent non-linear dynamics or introduce probabilistic variables into the model. On the other hand, the statistical methods used for evaluation of the effect of various factors on transmission are often based on specific types of data, meaning that they cannot identify patterns and relationships among other types of data; in addition, they are not generally suited to dealing with massive data.

This research study is motivated by the increasing availability of different types of data, as well as the availability of massive data, which naturally lend themselves to data-driven approaches for epidemic spread prediction [11,12,13,14]. Compared to the research studies described above, which employed known time-series forecasting models, or were predicated on a specific model, we propose the use of ordinal classification algorithms for the prediction and evaluation of the epidemic spread. These algorithms are designed to address the aforementioned limitations of previous models. The next section presents a review of classification algorithms and their adaptability for the evaluation of different factors affecting the spread of an epidemic—the goal of which is to predict the daily growth rate factor.

### 1.2. Classification Methods for the Evaluation of Different Factors Affecting the Spread of an Epidemic

Classification is one of the most common tasks in machine learning. It is used to identify to which of a set of classes a new observation belongs, based on the values of the explanatory or input variables. In our research, the classification task consists of distinguishing between different levels of a growth rate factor [15] that represents the epidemic spread. The classification is based on the identification of relationships among variables that represent specific conditions and risk factors. For data in which the class attribute exhibits some form of ordering (such as the growth factor level), ordinal classification can be applied, which takes into account the ranking relationship among classes [16]. Ordinal problems commonly address real-world applications such as portfolio investment by expected return performance or classification of the severity of disease, in which a classification error could have critical consequences [17,18,19,20,21]. Most of these techniques assume monotonicity between the explaining and target attributes [21,22,23,24,25]. Several previous research studies have shown that ordinal classifiers yield poor classification accuracy when applied to datasets with high levels of non-monotonic noise [26]. In recent studies, Singer et al. and Singer and Cohen [27,28] proposed an ordinal classification tree based on a weighted information gain measure. They found it to be effective for classification problems in which the class variable exhibits some form of ordering, and where dependencies between the attributes and the class value can be non-monotonic, as may be the case for the current problem of the control of epidemic spread based on environmental factors. For example, the ordinal attribute “forecast temperature” may have a non-monotonic effect on the growth factor; that is, extreme temperature conditions, either very high or very low, may lead to lower growth factor values, while under “moderate” temperatures, the growth factor may be higher. The weighted information gain measure proposed in these studies takes into consideration the magnitude of the potential classification error, where this error is calculated relative to the value of a specific class of the target. In this research paper, we extend the weighted information gain measure such that the classification error can be measured from a statistical value that is not necessarily defined by a single class—for example, the expected value of all classes. We use the proposed measure to develop ordinal decision-tree-based ensemble approaches, i.e., ordinal AdaBoost and random forest models, which are known to outperform individual classifiers. We demonstrate that these ordinal decision-tree-based approaches are naturally suited to identifying large numbers of data patterns with complex dependence structures, without requiring a priori assumptions regarding the dependencies within the data. Thus, the proposed algorithms have the precise characteristics required to address the aforementioned shortcomings of existing approaches for evaluating the effects of different factors on daily and regional growth factors of an epidemic.

The main objectives of this study are fourfold: (i) to extend the weighted information gain measure such that the classification error can be measured from a statistical value that is not necessarily defined by a single class; (ii) to develop ordinal decision-tree-based ensemble approaches in which an objective-based information gain measure is used; (iii) to examine the advantage of combining ordinal decision-tree-based ensemble approaches with non-ordinal individual classifiers to leverage the strengths of each type of classifier; and (iv) to examine the ability to carry out multi-class identification of different levels of a daily growth factor using ordinal decision-tree-based ensemble approaches.

The remainder of the paper is organized as follows. In Section 2, we provide a detailed description of the proposed ordinal decision-tree-based ensemble approaches. Section 3 presents numerical experiments for evaluating and benchmarking the proposed algorithms against known non-ordinal algorithms. The input data for these experiments consist of COVID-19 growth rate factors, along with environmental factors and containment measures for 19 regions in Italy. Finally, the conclusions and discussion are presented in Section 4.

## 2. Materials and Methods

In this section, we begin by presenting an extension to a general version of the objective-based entropy measure proposed by Singer et al. and Singer and Cohen [27,28] (Section 2.1). In Section 2.2, we develop ordinal AdaBoost and random forest algorithms based on ordinal decision tree models that use this measure. Further, we propose a majority voting approach based on combined ordinal and non-ordinal algorithms. We then continue, in Section 2.3, by describing the dataset used for classification of the daily COVID-19 growth rate factor, which will be used to evaluate the performance of the suggested approaches.

### 2.1. An Objective-Based Information Gain Measure for Ordinal Decision-Tree-Based Algorithms

The objective-based information gain (OBIG) is a measure for selecting the attributes with the greatest explanatory value in a decision tree model. The goal of our research is to identify the severity of the epidemic spreading process by classifying the level of the daily growth factor (DGF). The OBIG is appropriate for this purpose, since it takes into consideration the ordinal nature of the DGF level and the magnitude of the potential classification error. Assume that we wish to classify the level of the DGF based on a dataset $D=\left\{\left(x_{m},y_{m}\right),\;m=1,2,\ldots ,M\right\}$, where $x_{m}=\left[v_{m,1},v_{m,2},\ldots ,v_{m,K}\right]$ denotes a sample *m* in the dataset, defined by a vector of values for the *K* attributes A= {$A_{1},A_{2},\ldots ,A_{K}\}$, and $y_{m}$ denotes the value of the daily growth factor of sample *m*. We categorize the values of $y_{m}$ into *n* different severity levels denoted by the random variable $C\in \left\{c_{i},i=1,\ldots ,n\right\}$, where $c_{1}$ denotes the lowest daily growth rate and $c_{n}$ the highest daily growth rate. The new discretized daily growth rate variable (the target variable) is denoted by $Y=\left\{Y_{m},m=1,2,\ldots ,M\right\}$. We define values for the different levels of the daily growth rate, $V\in \left\{v(c_{i}),i=1,\ldots ,n\right\}$, as an increasing function of the severity, such that $v(c_{i})<v(c_{j}),\;\forall i<j$. The precise values of the classes should be assigned by considering the consequences of potential classification errors. For example, assume that we have three levels of daily growth rate, (*c*_1_, *c*_2_, *c*_3_) = (*low*, *medium*, *high*), and the decision-makers are conservative regarding restricting the movements of the public, as they wish to prevent medium and high spreading rates of the epidemic. Thus, when a “high” daily growth rate is predicted, the recommendation is global closure, while when a “medium” daily growth is predicted, the recommendation is local closures, which decision-makers believe can also be a highly effective strategy in the case of a high growth rate. It is only when a “low” daily growth rate is predicted that it is considered acceptable to remove all restrictions. The health consequences of predicting a low daily growth rate while the actual growth rate is medium are more severe than those of predicting a medium daily growth rate when the actual growth rate is high. Accordingly, we would assign values to the classes such that the relationship between the values of different levels is $v(c_{3})-v(c_{2})<v(c_{2})-v(c_{1})$. The OBIG uses an objective-based entropy (OBE) measure that, like the conventional concept of entropy, measures the randomness and uncertainty of the outcome of a random variable. However, unlike the conventional measure, the OBE allocates different weights to the classes as follows:

(1) $$\mathrm{OBE}\left(Y,T\right)=-\overset{n}{\underset{i=1}{\sum }}\omega \left(c_{i}\right)P\left(c_{i}\right)\log P\left(c_{i}\right),\;0\leq k\left(c_{i}\right)\leq 1,\;\mathrm{where}\;\overset{n}{\underset{i=1}{\sum }}\omega \left(c_{i}\right)=1,$$

where $P\left(c_{i}\right)$ is the probability that record *m* belongs to class $c_{i}$, and $\omega \left(c_{i}\right)$ is the weight of class $c_{i}$. In previous studies that used the OBE formula [27,28], the weights assigned to different classes were calculated according to the values and dispersions of the classes with respect to the value of selected class $C^{s}\left(Y,V\right)$ defined by statistic *s*. Among the selected classes proposed in these studies were the class with the maximum value, $c^{max}=\underset{c_{i}}{\arg\;\max\;V\left(c_{i}\right)}$, and the most probable class in the dataset, $c^{mode}=\underset{c_{i}}{\arg\;\max P\left(c_{i}\right)}$. In this research study, we generalize the OBE measure by replacing the value of the selected class, $v(C^{s}\left(Y,V)\right)$, with a targeted value, T(Y,V). The targeted value should not necessarily be related to a selected class, but can reflect a statistical property of the set of classes, such as its expected value:

(2) $$T\left(Y,V\right)=EV\left(Y,V\right)=\overset{n}{\underset{i=1}{\sum }}P\left(c_{i}\right)\cdot V\left(c_{i}\right).$$

Specific examples of the targeted value, as presented in previous studies, include $T\left(Y,V\right)=v(c^{max})$ and $T\left(Y,V\right)=v(c^{mode})$. Thus, we define the weight of class $c_{i}$ in this research study as

(3) $$\omega \left(c_{i}\right)=\frac{{\left|v\left(c_{i}\right)-T\left(Y,V\right)\right|}^{\alpha }}{\sum_{i=1}^{n}{\left|v\left(c_{i}\right)-T\left(Y,V\right)\right|}^{\alpha }}$$

where $\omega \left(c_{i}\right)$ is calculated as follows: the absolute deviation of the value of the *i*th class, $v\left(c_{i}\right)$, from the targeted value, T(Y,V), divided by the sum of all absolute difference values over all possible classes in the dataset. This measure implies that an attribute with a smaller distribution around the targeted value obtains a smaller OBE value, which represents a lower risk. The factor α (α>0) is a normalization factor that smooths the distribution of the weights over the different classes. The objective-based entropy measure is used for the calculation of an objective-based information gain measure, ${\mathrm{OBIG}}_{k}\left(D,T\right)$, for selecting branching attributes in dataset D in decision tree models by partitioning the records in D over the attribute $A_{k}$ having $N_{k}$ distinct values as follows:

(4) $$\mathrm{OBI}G_{k}\left(D,T\right)=\mathrm{OBE}\left(T,Y\right)-\overset{N_{k}}{\underset{r=1}{\sum }}\frac{\left|D_{k}^{r}\right|}{\left|D\right|}\times \mathrm{OBE}\left(Y_{k}^{r},T\right),$$

where the second expression on the right-hand side of the equation is the objective-based entropy of a possible partitioning on the attribute $A_{k}$. The value $\frac{\left|D_{k}^{r}\right|}{\left|D\right|}$ represents the weight of the *r*th partition of attribute $A_{k}$, i.e., the number of records in $D_{k}^{r}$ relative to the total number of records in *D*, and $\mathrm{OBE}\left(Y_{k}^{r},T\right)$ represents the objective-based entropy of the sub-dataset $Y_{k}^{r}\subseteq Y$. Similar to the conventional information gain measure, the objective-based information gain is overly sensitive to the number of distinct values $N_{k}$; thus, it should be normalized (at least for some algorithms) when there is a large variance in the number of values for different attributes [29,30,31]. In our research, we used the CART model as well as ordinal decision-tree-based ensemble approaches that are composed of CART models. Given that these algorithms branch via binary splitting at each node of the tree ($N_{k}=2,\;\forall k$), we used the OBIG measure in Equation (4) without normalization. The attribute $A_{k}$ with the highest (non-negative) weighted information gain was selected as the branching attribute of the node.

### 2.2. Incorporating the Objective-Based Information Gain Measure into Ensemble Methods

Ensemble methods attempt to overcome the bias or variance effects of individual classifiers by combining several of them together [32], thus achieving better performance [33,34]. Despite the fact that ensemble methods for nominal classification have received considerable attention in the literature and have been chosen in preference to single individual learning algorithms for many classification tasks, the use of ordinal classification in ensemble algorithms has rarely been discussed [35]. In the literature, the most widely used ensemble methods are categorized into four techniques: bagging, boosting, stacking, and voting [35,36]. In this research, we suggest integrating the objective-based information gain into ensemble methods to enable the use of ordinal classification in ensemble algorithms. Specifically, in Section 2.2.1, we propose an ordinal random forest (RF) algorithm to implement ordinal classification in an ensemble approach based on the bagging technique. In Section 2.2.2, we propose an ordinal AdaBoost algorithm, which is based on the boosting technique. In Section 2.2.3, we propose a majority voting approach based on a combination of non-ordinal and ordinal decision-tree-based models.

#### 2.2.1. Ordinal Random Forest

**Figure 1:**
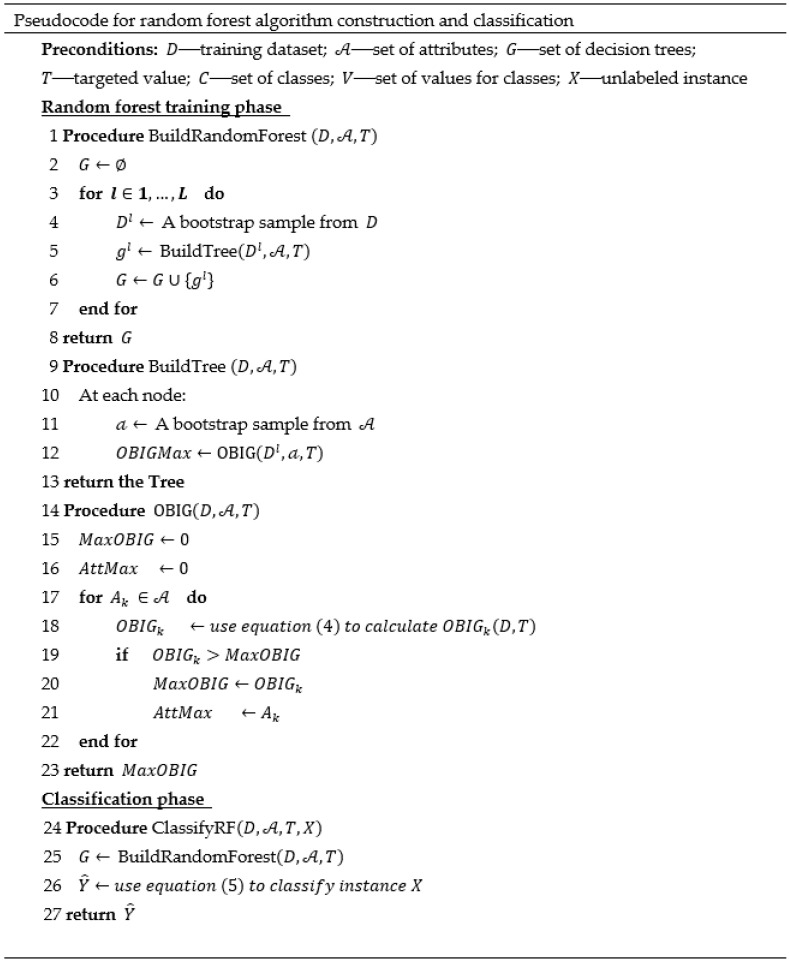
The meta-code of the proposed ordinal random forest classifier based on the objective-based information gain (OBIG) measure.

The random forest algorithm is a bagging method in which we create random sub-samples of our dataset with replacement, and we train decision trees on each sample. Since, in each sub-sample, the gain of each attribute with respect to the target variable may be similar, the different decision trees may have considerable structural similarity. Thus, in order to ensure that the trees are less correlated, while still ensuring that the samples are chosen randomly, the algorithm samples over the attributes in each node and uses only a random subset of them to choose the variable to split on, which reduces the similarities between different decision trees. A random forest built from several learners can significantly reduce the variance, i.e., fit the training data well, but can perform poorly with testing data (called overfitting), usually as a result of the model’s high complexity; thus, the RF approach is most suitable when combining individual learners when each of them suffers from large variance. Indeed, several studies have shown that random forests yield accurate and robust classification results when high variance exists [37,38,39]. Figure 1 presents the pseudocode for the construction of the random forest algorithm, as well as for the classification phase. In the construction phase, the random forest is built from *L* decision trees {$g_{l},l=1,2,\ldots ,L\}$, each trained on the *l*th bootstrap sample of the dataset, $D^{l}\subset D$. For each node in the tree, we randomly select a subset of attributes 𝒶⊂A, and the attribute with the highest objective-based information gain is selected as the splitting attribute using the procedure OBIG(D,A,T). In the classification phase, for each new instance X given to the decision tree classifiers as an input, the output $g^{l}\left(X\right)=c_{i}$ is returned by each model, and the class with the highest number of votes is chosen as follows:

(5) $$\hat{Y}=\underset{c_{i}\in C}{arg\;max}\overset{L}{\underset{l=1}{\sum }}\theta^{l}\left(X\right)$$

where

$$\theta^{l}\left(X\right)=\{\begin{array}{ll} 1, & if\;g^{l}\left(X\right)=c_{i}\\ 0, & otherwise. \end{array}$$

In the case where two classes receive the same number of votes, the algorithm chooses one of them at random.

#### 2.2.2. Ordinal AdaBoost

The AdaBoost algorithm is an adaptive boosting method in the sense that subsequent weak classifiers are tweaked in favor of those instances misclassified by previous classifiers, i.e., the classifiers are built sequentially and not in parallel like in the random forest algorithm. Each subsequent classifier focuses on previous “harder-to-classify” instances by increasing their weights in the dataset. In our example, we use 1-level decision trees based on the OBIG measure as the weak classifiers (low-complexity models). AdaBoost can significantly reduce the bias, i.e., the difference between the predicted value and true target value in the training data (called “underfitting”), which usually occurs due to low complexity of the model. Like the random forest approach, AdaBoost reaches a classification by applying multiple decision trees to every sample and combining the predictions made by individual trees. However, rather than selecting the class with the majority vote among the decision trees, as in random forest, in the AdaBoost algorithm, every decision tree contributes to a varying degree to the final prediction according to the incorrectly classified samples (as described below). Several studies have shown that AdaBoost yields accurate and robust results when high bias exists [40,41,42].

**Figure 2:**
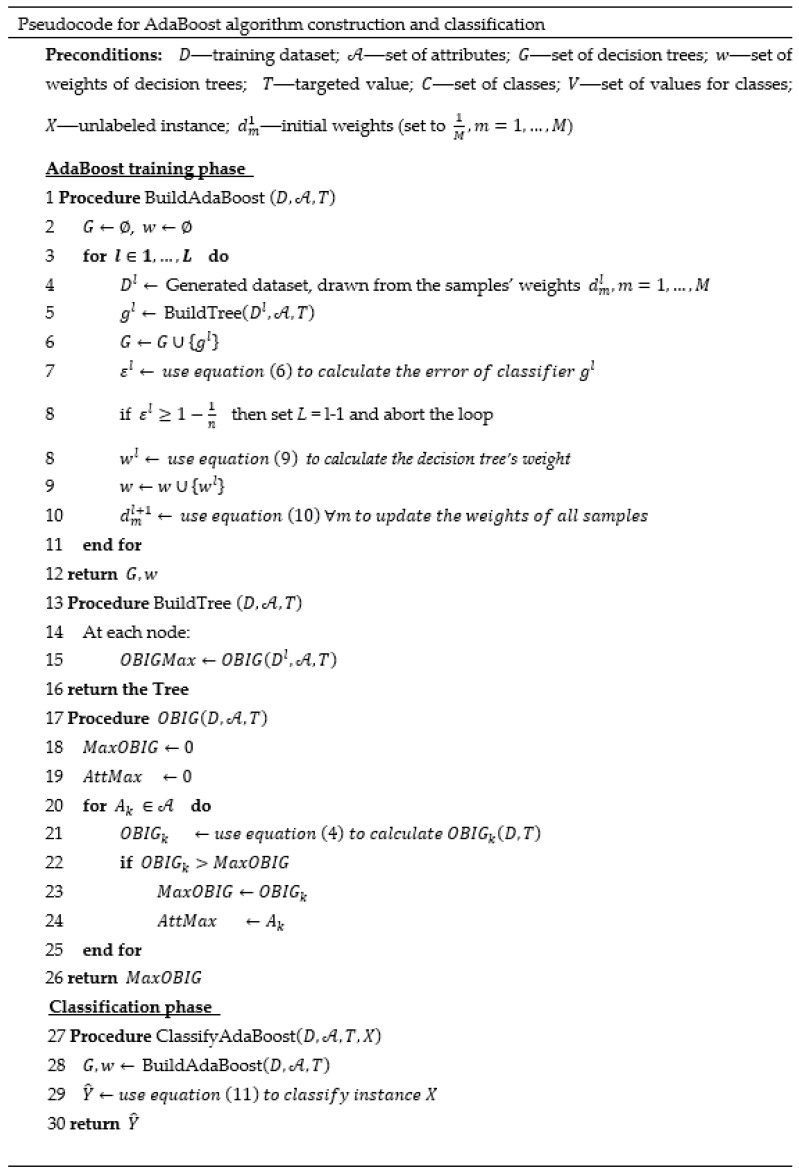
The meta-code of the proposed ordinal AdaBoost classifier based on the OBIG measure.

Figure 2 presents the pseudocode for the construction and classification phases of the AdaBoost model built from 1-level decision trees based on the OBIG measure. In the construction phase, the BuildAdaBoost (D,A,T) procedure builds *L*-many decision trees, each with a depth of 1 ({$g_{l},l=1,2,\ldots ,L\}$). These are built sequentially, where the goal is for each tree to improve upon its predecessor by being trained on a dataset $D^{l}$, generated from a set of sample weights $d_{m}^{l},\;m=1,\ldots ,M$, such that samples that were misclassified by previous decision trees have higher weights. We begin in the first step, *l* = 1, by building a decision tree under the assumption of identical weights for the *M* samples in the dataset, $d_{m}^{1}=\frac{1}{M},\;m=1,\ldots ,M$. At the end of each iteration, we calculate the classifier error and use it to update the samples’ weights for the subsequent iteration and to determine the weight of the current decision tree in the final classification. The error of the classifier is calculated as follows:

(6) $$\varepsilon^{l}=\overset{M}{\underset{m=1}{\sum }}d_{m}^{l}e_{m}^{l},$$

where $e_{m}^{l}$ is the error function, which is defined in the AdaBoost algorithm by

(7) $$e_{m}^{l}=\{\begin{array}{ll} 1, & if\;g^{l}\left(x_{m}\right)\neq y_{m}\\ 0, & otherwise. \end{array}$$

Thus, the error is assigned a value of 1 when the predicted value $g^{l}\left(x_{m}\right)$ of a sample $x_{m}$ is different from the real value, and 0 otherwise. Note that we require $0\leq \varepsilon^{l}<1-\frac{1}{n}$, such that if the constraint does not hold, we stop generating decision trees. The expression $1-\frac{1}{n}$ reflects the error of a naïve classifier (i.e., random choice of a class) assuming equal probabilities for the classes. Note that Equation (7) assumes the same error value of 1 for each incorrectly classified sample, regardless of the magnitude of the classification error. Thus, an alternative option for an error function that considers the magnitude of the classification error would be

(8) $$e_{m}^{l}=\frac{\left|v\left(g^{l}\left(x_{m}\right)\right)-v\left(y_{m}\right)\right|}{\underset{i,j}{max}\left|v\left(c_{i}\right)-v\left(c_{j}\right)\right|},$$

such that the error value is the difference between the values of the predicted and actual class divided by the maximum difference between two different class values, where the denominator serves to normalize the error to the range $0\leq e_{m}^{l}\leq 1$. As mentioned, we use the error $e_{m}^{l}$ to calculate the decision tree weight in the final classification, which is achieved as follows:

(9) $$w^{l}=\ln\left(\frac{1-\varepsilon^{l}}{\varepsilon^{l}}\right)+\ln\left(n-1\right),$$

where $w^{l}>0$, and its value increases as the classifier’s error decreases, while $w^{l}\to 0^{+}$ when $\varepsilon^{l}\to 1-\frac{1}{n}$, which means that the weight of a classifier tends to zero as its error tends to the naïve classifier’s error. We also use $w^{l}$ to update the samples’ weights, such that misclassified samples obtain higher weights as follows:

(10) $$d_{m}^{l+1}=\frac{d_{m}^{l}}{Z^{l}}e^{\left(w^{l}e_{m}^{l}\right)},\;\forall m,$$

where $Z^{l}=\sum_{m=1}^{M}d_{m}^{l}e^{\left(w^{l}e_{m}^{l}\right)}$ is a normalization constant such that the new sample weights will sum up to 1. Note that when the error of the classifier tends to the naïve classifier error, $\varepsilon^{l}\to 1-\frac{1}{n}$ (i.e., $w^{l}\to 0^{+}$), or when the error is equal to zero, $\varepsilon^{l}=0$ (i.e., all samples classified correctly), then the weights of the samples will not change compared to previous iterations up to a constant. In any other case, $d_{m}^{l}$ decreases for samples that were correctly classified.

In the classification phase, for each new instance X given to the decision tree models as an input, the output $g^{l}\left(X\right)=c_{i}$ is returned by each model and contributes $w^{l}$ to the final prediction as follows:

(11) $$\hat{Y}=\underset{c_{i}\in C}{arg\;max}\overset{L}{\underset{l=1}{\sum }}w^{l}\;\theta^{l}\left(X\right),$$

where $\theta^{l}\left(X\right)$ is defined in Equation (5).

#### 2.2.3. Ensemble Approach Based on Decision-Tree-Based Algorithms

In order to benefit maximally from the various ordinal decision-tree-based algorithms (each with its own objective-based entropy measure), as well as from non-ordinal algorithms, we propose a simple ensemble approach, which, in theory, should leverage the strengths of each individual classifier [43]. Assume that we wish to build *J*-many classifiers $\left\{G^{j},j=1,2,\ldots ,J\right\}$. For each new sample X given to classifier j as an input, the output ${\hat{Y}}_{j}$ is returned using the classification procedure of the relevant model, and the class with the highest number of votes is chosen as follows:

(12) $$\underset{c_{i}\in C}{arg\;max}\overset{J}{\underset{j:{\hat{Y}}_{j}=c_{i}}{\sum }}1.$$

### 2.3. The Dataset and Data Preparation

We constructed a COVID-19 dataset (see Appendix A) to evaluate the newly developed algorithms and to compare them with their non-ordinal counterparts and other conventional algorithms. The dataset is based on 43 features, created from three different types of daily data relating to 19 regions of Italy between 7th March and 1st April 2020. (The region and the date are two additional features of the dataset, which we refer to as “key features”). The three types of data are as follows: (i) *COVID-19 patient data* [44], which consists of 13 numerical features (e.g., number of new positive cases, number of tests performed); (ii) *weather data* [45], comprising 15 numerical features (e.g., temperature, humidity, pressure, wind speed); and (iii) *containment and mitigation measures data* [46], which includes 15 Boolean features (e.g., regulations regarding outdoor gatherings, public transport cleaning, mass isolation, and school closure). Our target value is the daily growth factor ($DGF_{t}$) of positive cases $O_{t}$ normalized by the number of tests performed, $\eta_{t}$, relative to the corresponding values for the previous day:

(13) $$DGF_{t}=\frac{\left(O_{t}/\eta_{t}\right)}{\left(O_{t-1}/\eta_{t-1}\right)}.$$

Daily growth rates equal to zero for specific regions and dates were removed from the dataset, since they usually reflect pre-COVID19-spread periods for these regions. In order to prepare the data for classification, we discretized the *DFG* into three different levels, denoted by $C=\left\{c_{1},c_{2},c_{3}\right\}$, where $c_{1}$ represents negative growth and is defined as DGF<0.9 (43% of cases), $c_{2}$ represents linear growth and is defined as DGF∈[0.9,1.1] (23% of cases), and $c_{3}$ represents exponential growth and is represented by DGF>1.1 (34% of cases). We denote the values for the different levels of the daily growth rate by $V\left(c_{i}\right)=i$. During this experiment, we aimed to predict at time τ=t the growth rate per region 6 days later (i.e., at time τ=t+6), based on (a) the last 6 days of *COVID-19 patient data* (i.e., data corresponding to τ∈[t−5,t]); (b) the subsequent 5 days of *weather data*, as forecast at time *t* (i.e., τ∈[t+1,t+5]); and (c) *containment measures* at time *t*. We assumed an incubation period of up to 5 days and the ability to forecast the weather 5 days in advance with high accuracy. We further assumed that only the containment measures for the current date were known (we observed from the data that, in most cases, the containment measures did not change during the following 5 days). After the preprocessing stage, the final dataset that was used as an input for the classification algorithms, $D=\left\{\left(x_{m},y_{m}\right),m=1,2,\ldots ,M\right\}$, consisted of M=463 samples and K=161 attributes, A= {$A_{1},A_{2},\ldots ,A_{K}\}$. For model evaluation, the data were split into a training dataset, corresponding to data for 7 March to 29 March (80% of the data), and a testing dataset of data for 28 March to 1 April (20% of the data). In that way, the model was trained on past data and then validated on future data that it had not previously encountered.

## 3. Results

### 3.1. A Comparison between Ordinal CART Classifiers and the Popular Non-Ordinal CART Classifier

This subsection compares the performance of the OBIG-based ordinal CART, i.e., a single decision tree, with the popular non-ordinal CART. Four different versions of the ordinal CART are evaluated, corresponding to four different targeted values, $T\left(Y,V\right)\in \left\{v(c^{max}),v(c^{mode}),v(c^{min}),\;EV\right\}$. For benchmarking purposes, the performance of the ordinal and non-ordinal classifiers was computed using three performance measures for multi-class classification: *F-score*, *Accuracy*, and *area under the curve (AUC)* [47]. Additionally, we used the *mean square error* (*MSE*) and *Kendall’s correlation coefficient*, $\tau_{b}$, which are acceptable performance measures for ordinal classification [27,48]. The best performance values are highlighted in bold in Table 1. The following insights can be gleaned from the table: (1) The ordinal CART algorithms based on $v(c^{max})$ and $v(c^{mode})$ yielded better performance than the regular non-ordinal CART in four out of five indices, while the versions based on EV and $v(c^{min})$ were superior for all indices. (2) The common classification performance measures (*F-score*, *Accuracy*, and *AUC*) were between 9% and 17% higher for the CART based on EV than for the non-ordinal CART. A similar improvement in performance is seen for *MSE*, while the improvement for $\tau_{b}$ is much higher.

**Figure 3:**
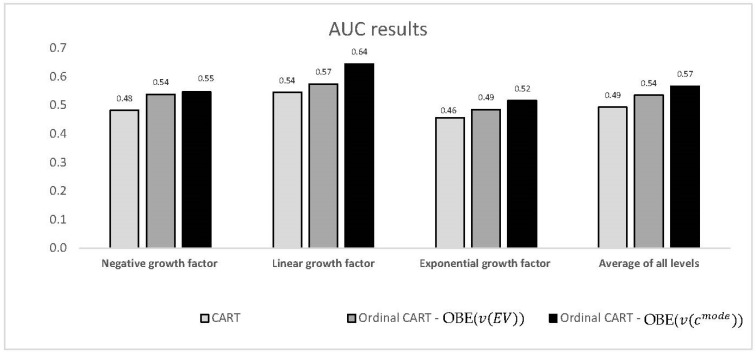
Comparison of the AUC values (*y*-axis) for two best ordinal CART models vs. non-ordinal CART as a function of the growth factor level (*x*-axis).

Figure 3 illustrates the *AUC* values obtained for each growth factor level, as well as the global *AUC*, for the two best ordinal models and the conventional non-ordinal CART model. It can be seen that the ordinal CART models yielded significantly better results for all classes than the individual classifier. The improvement in performance for the best ordinal CART model ranges from 13% to 19% (for the three individual levels and the global average).

### 3.2. A Comparison between Ordinal and Non-Ordinal Ensemble-Based Classifiers

This subsection reports the performance of the OBIG-based ordinal AdaBoost and random forest algorithms (Table 2 and Table 3, respectively) and compares them with their non-ordinal ensemble counterparts. The best performance values in each table are highlighted in bold. We observe the following: (1) All ordinal AdaBoost algorithms except for the one based on OBE($v(c^{mode})$) achieved better performance than the conventional AdaBoost with respect to all five indices. (2) All ordinal random forest algorithms outperformed their non-ordinal counterpart for all indices, with the exception of the ordinal random forest based on OBE(EV), which yielded a lower value for the Kendall index. (3) The ordinal AdaBoost model based on $\mathrm{OBE}\left(v(c^{max}\right))$ and the ordinal random forest model based on OBE($v(c^{mode})$) yielded the best performance for all five indices. (4) The ranges of improvement of the best ordinal AdaBoost and random forest classifiers relative to their non-ordinal counterparts for the classification measures *F-score*, *Accuracy*, and *AUC* are 14–25% and 6–21%, respectively. The improvement in performance is similar for *MSE* and considerably higher for $\tau_{b}$.

**Figure 4:**
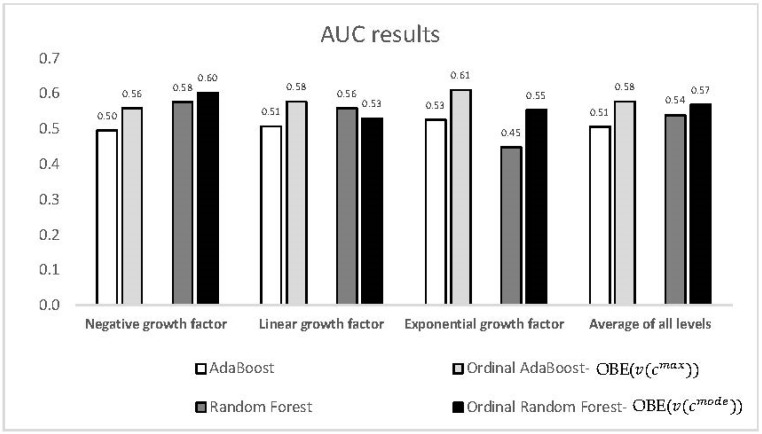
Comparison of the AUC values (*y*-axis) for the best ordinal AdaBoost and random forest classifiers vs. their conventional counterparts as a function of the growth factor level (*x*-axis).

Figure 4 illustrates the *AUC* values obtained for each growth factor level for the best ordinal AdaBoost and random forest classifiers compared to their conventional non-ordinal counterparts. It can be seen that the ordinal models yielded significantly better results for all classes except for the case of linear growth in the AdaBoost models. However, errors relative to fringe classes usually lead to more serious consequences; thus, in ordinal problems, it is more important to achieve better performance for these classes.

### 3.3. A Comparison between the Predictions of Ordinal Classifiers and Non-Ordinal Classifiers

In this section, we evaluate whether the differences between the predictions of some of the best ordinal classifiers and their non-ordinal counterparts are significant. This analysis was carried out for each of the three types of classifier—CART, AdaBoost, and random forest—by conducting a two-tailed paired-samples t-test using pairs of predictions for each instance in the testing dataset. Table 4 summarizes the results, showing that the difference was found to be significant for the ordinal decision-tree-based ensemble approaches (*p* < 0.05).

**Figure 5:**
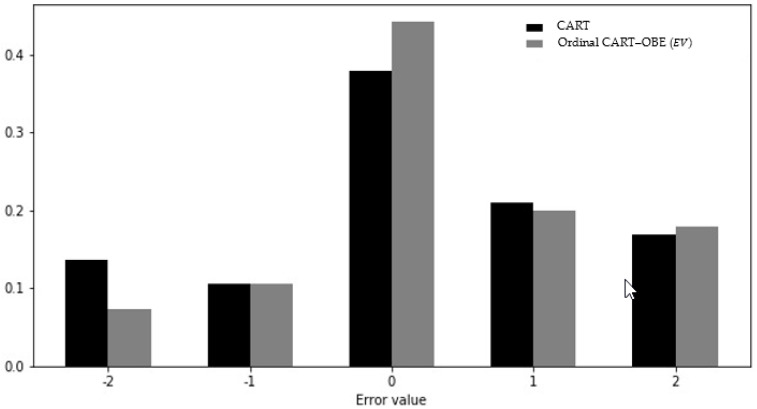
Error distribution of ordinal CART and non-ordinal CART.

**Figure 6:**
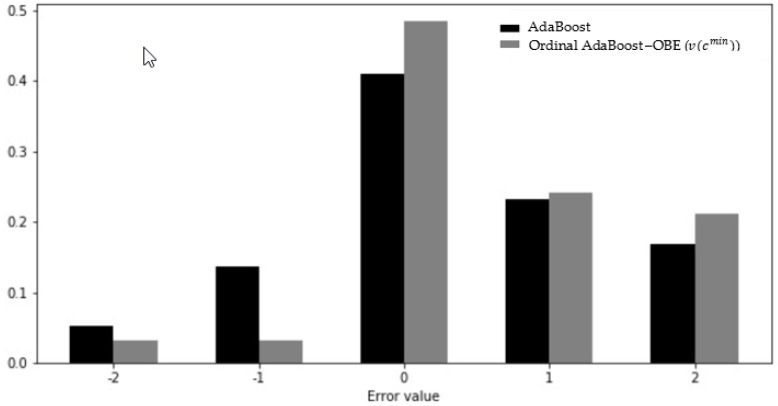
Error distribution of ordinal AdaBoost and non-ordinal AdaBoost.

**Figure 7:**
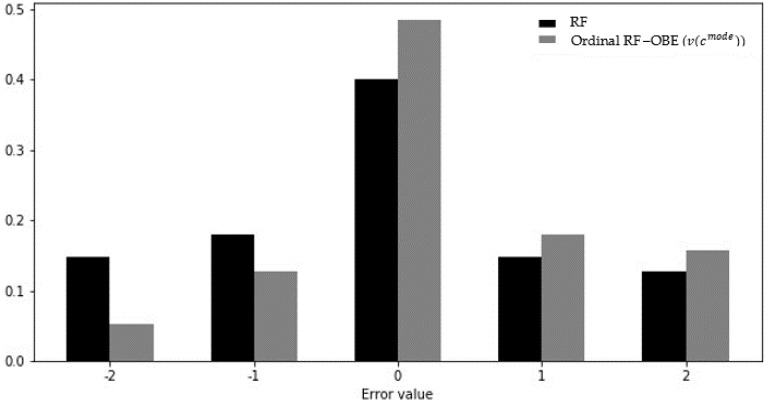
Error distribution of ordinal random forest and non-ordinal random forest.

Figure 5, Figure 6 and Figure 7 present the distributions of the errors (the actual class values minus the predicted class values) for the ordinal classifiers compared to their non-ordinal counterparts. It can be seen that the frequencies of zero-error cases for the ordinal algorithms were substantially higher than for their non-ordinal counterparts. Furthermore, the frequency of errors with a value of −2 (i.e., the prediction of an exponential growth factor when the actual growth factor was negative) was higher in all non-ordinal models than in the corresponding ordinal models, with the difference being at least a factor of 2 in the case of the CART and random forest models.

### 3.4. A Comparison between Ordinal Classifiers and Non-Ordinal Classifiers

For benchmarking purposes, in this subsection we compare the best ordinal classifiers from the previous analysis with a number of popular non-ordinal classifiers. Table 5 presents the performance measures for eleven individual classifiers (eight non-ordinal classifiers and three ordinal), with the two best performance values highlighted in bold. The following insights can be gleaned from the table: (1) The ordinal AdaBoost yielded the best results for *F-score*, *Accuracy*, and *AUC*, and among the two best results for *MSE* and $\tau_{b}$. Overall, it appears to be the best among the individual classifiers. (2) Logistic regression and ordinal random forest are among the two best classifiers with respect to two indices.

### 3.5. Ensemble Majority Voting Approach Based on Ordinal Classifiers

In this subsection, we apply a majority voting ensemble approach based on ordinal and non-ordinal classifiers. We combined the ordinal AdaBoost with targets $T\left(Y,V\right)\in \left\{v(c^{max}),EV\right\}$, which were found to be the best AdaBoost classifiers in previous analyses, with the best non-ordinal classifier—logistic regression. The performance of the majority voting model is reported in the first column of Table 6. For comparison, we also show the performance of two individual classifiers—logistic regression (Column 2) and the best ordinal classifier, AdaBoost based on $v(c^{max})$ (Column 3). The best results are indicated in bold. The majority voting model outperformed the individual classifiers for all five indices with the ranges of improvement for *Accuracy*, *AUC*, and *F-score* being 3–6% (relative to the ordinal classifier) and 6–10% (relative to the non-ordinal classifier).

## 4. Conclusions and Discussion

In this research we suggest an extension to the objective-based information gain (OBIG) measure that was proposed in [27,28] for selecting the attributes with the greatest explanatory value in a classification problem. In these studies, the weights assigned to different classes were calculated with respect to the value of a selected class. In the present study, we introduced a general targeted value function that is not necessarily related to a specific class. Based on the extended OBIG measure, we proposed novel, ensemble-based ordinal approaches, i.e., (1) ordinal AdaBoost and ordinal random forest decision tree models and (2) a majority voting approach that combines these models together with conventional non-ordinal algorithms. We demonstrated how the ensemble ordinal approaches may be implemented to evaluate the effect of different factors on the level of the regional daily growth factor (DGF) of the spread of an epidemic in order to yield a classification value. The construction of the proposed models considers the magnitude of the classification error of the daily growth factor. The classification tool will enable the spreading process to be tracked and controlled, as the models can yield insights regarding the link between local containment measures and the DGF.

We evaluated the performance of the suggested approaches for classification of the daily COVID-19 growth rate factor in 19 regions of Italy. A comparison of each of the ordinal models with its conventional, non-ordinal counterpart demonstrated that the proposed models are superior based on a variety of common performance metrics for both conventional and ordinal classification problems. Specifically, the best individual ordinal CART model yielded a 9–17% improvement when compared to the conventional CART model for three common indices for conventional classification problems: *F-score*, *Accuracy*, and *AUC*. For the same indices, the best ordinal AdaBoost model yielded a 14–25% improvement when compared to the conventional AdaBoost model, and the ordinal random forest model yielded a 6–21% improvement when compared to its non-ordinal counterpart. A similar level of improvement was observed for one of the performance measures designed for ordinal classification (*MSE*), while the second such measure (*Kendall’s correlation coefficient*) showed much greater improvement. Furthermore, the ordinal AdaBoost was shown to be the best individual classifier when compared to all other ordinal classifiers and eight popular non-ordinal classifiers. Finally, we investigated a majority voting approach that combines ordinal and non-ordinal classifiers. This ensemble approach achieved better performance in all indices than the best individual ordinal and non-ordinal classifiers, with a level of improvement of 3–10% in all indices.

The level of improvement offered by the proposed ordinal approaches relative to their non-ordinal counterparts suggests that these approaches show promise for classification of the regional daily growth factor level in the spread of an epidemic, which is an ordinal target problem with no monotonic constraints on the explaining attributes. However, despite the fact that in our experiment, the relative improvement between the ordinal and the non-ordinal models is systematic, the experiment was performed only on a single dataset with a relatively low number of instances (which limits the prediction performance of the models), while ensemble models are mostly suitable for datasets with large numbers of instances.

In conclusion, the main findings of this study are as follows. First, the ordinal decision-tree-based ensemble approaches yielded better classification results than their non-ordinal counterparts, and the best ordinal classifier outperformed eight popular non-ordinal classifiers. Second, when implementing an ensemble approach by combining two ordinal decision tree algorithms with a non-ordinal algorithm, the classification performance is improved even further. Third, the proposed approaches are suitable for carrying out multi-class identification of different levels of the daily growth factor rate. Future research could apply the suggested ordinal algorithms to other datasets, including datasets with larger numbers of instances, to verify the robustness of the algorithms with respect to different settings. Future studies could also consider integrating the magnitude of the classification error into other boosting ensemble methods, such as gradient boosting or XGBoost algorithms. Furthermore, it could be interesting to examine the performance of other ensemble approaches, such as stacking or soft voting.

## Figures and Tables

**Table 1:** Performance measures of CART models for the classification of the daily growth factor.

	Performance Measures for Classification			Performance Measures for Ordinal Classification	
	*F-Score*	*Accuracy*	*AUC*	*MSE*	$\tau_{b}$
**Non-ordinal classifier**					
CART	0.361	0.379	0.493	1.537	−0.079
**Ordinal classifiers**					
Ordinal CART–OBE($v(c^{mode})$)	0.391	0.389	**0.567**	1.684	−0.011
Ordinal CART–OBE($v(c^{max})$)	0.366	0.358	0.518	**1.211**	0.068
Ordinal CART–OBE($v(c^{min})$)	0.385	0.389	0.526	1.274	**0.090**
Ordinal CART–OBE(EV)	**0.409**	**0.442**	0.535	1.316	0.016

**Table 2:** Performance measures for the classification of the daily growth factor using ordinal and non-ordinal AdaBoost models.

	Performance Measures for Classification			Performance Measures for Ordinal Classification	
	*F-Score*	*Accuracy*	*AUC*	*MSE*	$\tau_{b}$
**Non-ordinal ensemble classifier**					
ADABoost	0.380	0.411	0.507	1.253	0.006
**Ordinal AdaBoost classifies**					
Ordinal AdaBoost–OBE($v(c^{mode})$)	0.377	0.381	0.504	1.56	−0.093
Ordinal AdaBoost–OBE($v(c^{max})$)	**0.475**	**0.526**	**0.578**	**1.137**	**0.163**
Ordinal AdaBoost–OBE($v(c^{min})$)	0.405	0.484	0.538	1.242	0.072
Ordinal AdaBoost–OBE(EV)	0.447	0.474	0.563	1.221	0.122

**Table 3:** Performance measures for the classification of the daily growth factor using ordinal and non-ordinal random forest models.

	Performance Measures for Classification			Performance Measures for Ordinal Classification	
	*F-Score*	*Accuracy*	*AUC*	*MSE*	$\tau_{b}$
**Non-ordinal ensemble classifier**					
Random forest (RF)	0.405	0.400	0.540	1.421	0.035
**Ordinal random forest classifiers**					
Ordinal RF–OBE($v(c^{mode})$)	**0.439**	**0.484**	**0.570**	**1.147**	**0.185**
Ordinal RF–OBE($v(c^{max})$)	0.407	0.453	0.559	1.400	0.126
Ordinal RF–OBE($v(c^{min})$)	0.425	**0.484**	0.557	1.211	0.131
Ordinal RF–OBE(EV)	0.437	0.442	0.555	1.411	0.026

**Table 4:** Paired t-test results for the significance of the difference in the predictions of the ordinal classifiers and their non-ordinal counterparts (ordinal CART vs. CART; ordinal AdaBoost vs. AdaBoost; ordinal random forest vs. random forest).

	Paired *t*-Test *p*-Value		
	Ordinal CART—OBE(EV)	Ordinal AdaBoost—OBE($v(c^{min})$)	Ordinal RF—OBE($v(c^{mode})$)
Non-ordinal counterpart	0.14	0.0057	0.0015

**Table 5:** Performance measures of the best ordinal classifiers in comparison to eight popular non-ordinal classifiers.

	Performance Measures for Classification			Performance Measures for Ordinal Classification	
	*F-Score*	*Accuracy*	*AUC*	*MSE*	$\tau_{b}$
**Non-ordinal classifiers**					
Naïve Bayes	0.246	0.305	0.478	**0.916**	−0.065
Logistic regression	**0.453**	**0.505**	0.560	1.189	0.060
Gradient boosting	0.347	0.356	0.481	1.611	−0.117
XGBoost	0.378	0.389	0.506	1.558	−0.077
K-nearest neighbor	0.433	0.453	0.543	1.305	0.013
AdaBoost	0.380	0.411	0.507	1.253	0.006
Random forest	0.405	0.400	0.540	1.421	0.035
CART	0.361	0.379	0.493	1.537	−0.079
**Ordinal classifiers**					
Ordinal CART–OBE(EV)	0.409	0.442	0.535	1.316	0.016
Ordinal AdaBoost—OBE($v(c^{max})$)	**0.475**	**0.526**	**0.578**	**1.137**	**0.163**
Ordinal RF—OBE($v(c^{mode})$)	0.439	0.484	**0.570**	1.147	**0.185**

**Table 6:** Comparison of global performance measures for the majority voting ensemble approach and the best individual classifiers (non-ordinal and ordinal).

Performance Measure	Majority Voting Model Based on Ordinal and Non-Ordinal Classifiers	Best Non-Ordinal Classifier: Logistic Regression	Best ordinal classifier: ordinal AdaBoost based on OBE$\left(v(c^{max})\right)$
*F-score*	**0.501**	0.453	0.475
*Accuracy*	**0.558**	0.505	0.526
*AUC*	**0.596**	0.560	0.578
*MSE*	**1.074**	1.189	1.137
$\tau_{b}$	**0.200**	0.060	0.016

## Appendix A. Full Dataset Used as an Input to the Classification Models

**Category**	**Fields**	**Field Type(s)**
Key fields	date, region	Date, Categorical
Italy’s COVID-19 patient data	latitude, longitude, hospitalized with symptoms, intensive care patients, total hospitalized patients, people in home isolation, current positive cases, change in total positive, new currently positive, discharged people healed, deceased people, total positive cases, tests performed	Numerical
Weather	max temperature (°F), average (avg) temperature (°F), min temperature (°F), max dew point (°F), avg dew point (°F), min dew point (°F), max humidity (%), avg humidity (%), min humidity (%), max wind speed (mph), avg wind speed (mph), min wind speed (mph), max pressure (Hg), avg pressure (Hg), min pressure (Hg)	Numerical
Containment	change in guidelines for reporting cases, basketball league suspension, public transport cleaning, proactively checking symptomatic patients, decree to lock down, outdoor gatherings banned, religious activity cancellation, massive cluster isolation, massive school closure, domestic travel limitation, sports cancellation, university closure, social distancing, limiting overload of supermarkets by customers, massive police patrol	Boolean
Target	daily growth rate	Categorical (ordinal)

## References

[1] Yang Z., Zeng Z., Wang K., Wong S.S., Liang W., Zanin M., Liu P., Cao X., Gao Z., Mai Z. (2020). Modified SEIR and AI prediction of the epidemics trend of COVID-19 in China under public health interventions. J. Thorac. Dis..

[2] Wang H., Wang Z., Dong Y., Chang R., Xu C., Yu X., Zhang S., Tsamlag L., Shang M., Huang J. (2020). Phase-adjusted estimation of the number of coronavirus disease 2019 cases in Wuhan, China. Cell Discov..

[3] Chen T.M., Rui J., Wang Q.P., Zhao Z.Y., Cui J.A., Yin L. (2020). A mathematical model for simulating the phase-based transmissibility of a novel coronavirus. Infect. Dis. Poverty.

[4] Chen T., Rui J., Wang Q., Zhao Z., Cui J.A., Yin L. (2020). A mathematical model for simulating the transmission of Wuhan novel Coronavirus. bioRxiv.

[5] Getz W.M., Salter R., Mgbara W. (2019). Adequacy of SEIR models when epidemics have spatial structure: Ebola in Sierra Leone. Philos. Trans. Royal Soc. B.

[6] Kramer A.M., Tomlin Pulliam J., Alexander L.W., Park A.W., Rohani P., Drake J.M. (2016). Spatial spread of the West Africa Ebola epidemic. Open Sci..

[7] Getz W.M., Salter R., Lyons A.J., Sippl-Swezey N. (2015). Panmictic and clonal evolution on a single patchy resource produces polymorphic foraging guilds. PLoS ONE.

[8] Mecenas P., Bastos R., Vallinoto A., Normando D. (2020). Effects of temperature and humidity on the spread of COVID-19: A systematic review. MedRxiv.

[9] Pedersen M.G., Meneghini M. (2020). Quantifying undetected COVID-19 cases and effects of containment measures in Italy. Preprint.

[10] Mastrandrea R., Barrat A. (2016). How to estimate epidemic risk from incomplete contact diaries data?. PLoS Comput. Biol..

[11] Feng Y., Wang B.C. (2019). A unified framework of epidemic spreading prediction by empirical mode decomposition-based ensemble learning techniques. IEEE Trans. Comput. Soc. Syst..

[12] Shi B., Zhong J., Bao Q., Qiu H., Liu J. EpiRep: Learning node representations through epidemic dynamics on networks. Proceedings of the 2019 IEEE/WIC/ACM International Conference on Web Intelligence (WI).

[13] Teng Y., Bi D., Guo X., Paul R. Predicting the Epidemic Potential and Global Diffusion of Mosquito-Borne Diseases Using Machine Learning.

[14] Chekol B.E., Hagras H. Employing machine learning techniques for the malaria epidemic prediction in Ethiopia. Proceedings of the 10th Computer Science and Electronic Engineering (CEEC).

[15] Ma J. (2020). Estimating epidemic exponential growth rate and basic reproduction number. Infect. Dis. Model..

[16] Frank E., Hall M. (2001). A simple approach to ordinal classification. Proceedings of the 12th European Conference on Machine Learning.

[17] Gaudette L., Japkowicz N. (2009). Evaluation methods for ordinal classification. Proceedings of the Canadian Conference on Artificial Intelligence.

[18] Cardoso J.S., Sousa R. (2011). Measuring the performance of ordinal classification. Int. J. Pattern Recognit. Artif. Intell..

[19] Destercke S., Yang G. (2014). Cautious ordinal classification by binary decomposition. Proceedings of the Joint European Conference on Machine Learning and Knowledge Discovery in Databases.

[20] Gutierrez P.A., Perez-Ortiz M., Sanchez-Monedero J., Fernandez-Navarro F., Hervas-Martinez C. (2015). Ordinal regression methods: Survey and experimental study. IEEE Trans. Knowl. Data Eng..

[21] Verbeke W., Martens D., Baesens B. (2017). RULEM: A novel heuristic rule learning approach for ordinal classification with monotonicity constraints. Appl. Soft Comput..

[22] Ben-David A., Sterling L., Pao Y.-H. (1989). Learning and classification of monotonic ordinal concepts. Comput. Intell..

[23] Ben-David A. (1995). Monotonicity maintenance in information-theoretic machine learning algorithms. Mach. Learn..

[24] Christophe M., Petturiti D. (2013). Monotone classification with decision trees. Proceedings of the 8th Conference of the European Society for Fuzzy Logic and Technology (EUSFLAT-13).

[25] Zhu H., Tsang E.C., Wang X.-Z., Ashfaq R.A.R. (2017). Monotonic classification extreme learning machine. Neurocomputing.

[26] Ben-David A., Sterling L., Tran T. (2009). Adding monotonicity to learning algorithms may impair their accuracy. Expert Syst. Appl..

[27] Singer G., Anuar R., Ben-Gal I. (2020). A weighted information-gain measure for ordinal classification trees. Expert Syst. Appl..

[28] Singer G., Cohen I. (2020). An objective-based entropy approach for interpretable models in support of human resource management: The case of absenteeism at work. Entropy.

[29] Singer G., Golan M., Rabin N., Kleper D. (2020). Evaluation of the effect of learning disabilities and accommodations on the prediction of the stability of academic behaviour of undergraduate engineering students using decision trees. Eur. J. Eng. Educ..

[30] Singer G., Golan M. (2019). Identification of subgroups of terror attacks with shared characteristics for the purpose of preventing mass-casualty attacks: A data-mining approach. Crime Sci..

[31] Moral-García S., Castellano J.G., Mantas C.J., Montella A., Abellán J. (2019). Decision tree ensemble method for analyzing traffic accidents of novice drivers in urban areas. Entropy.

[32] Zhou Z.H. (2009). Ensemble Learning. Encycl. Biom..

[33] Bishop C.M. (1995). Neural Networks for Pattern Recognition.

[34] Kittler J., Hatef M., Duin R.P.W., Matas J. (1998). On combining classifiers. IEEE Trans. Pattern Anal. Mach. Intell..

[35] Yıldırım P., Birant U.K., Birant D. (2019). EBOC: Ensemble-based ordinal classification in transportation. J. Adv. Transp..

[36] Liang D., Tsai C.F., Dai A.J., Eberle W. (2018). A novel classifier ensemble approach for financial distress prediction. Knowl. Inf. Syst..

[37] Sathyadevan S., Nair R.R., Behera H.S., Mohapatra D.P. (2016). Comparative analysis of decision tree algorithms: ID3, C4.5 and random forest. Computational Intelligence in Data Mining—Volume 1, Proceedings of the International Conference on CIDM, 5–6 December 2015.

[38] Belgiu M., Drăguţ L. (2016). Random forest in remote sensing: A review of applications and future directions. ISPRS J. Photogramm. Remote Sens..

[39] Masetic Z., Subasi A. (2016). Congestive heart failure detection using random forest classifier. Comput. Methods Programs Biomed..

[40] Wang Y., Han P., Lu X., Wu R., Huang J. The performance comparison of Adaboost and SVM applied to SAR ATR. Proceedings of the 2006 CIE International Conference on Radar.

[41] Vezhnevets A., Vezhnevets V. (2005). Modest AdaBoost—Teaching AdaBoost to generalize better. Graphicon.

[42] Sun B., Chen S., Wang J., Chen H. (2016). A robust multi-class AdaBoost algorithm for mislabeled noisy data. Knowl.-Based Syst..

[43] Alpaydin E. (2020). Introduction to Machine Learning.

[44] Kumar S. Covid19 in Italy. https://www.kaggle.com/sudalairajkumar/covid19-in-italy.

[45] The Weather Channel, Wunderground The Weather Company, an IBM Business. https://www.wunderground.com.

[46] Epidemic Forecasting Global NPI (EFGNPI). http://epidemicforecasting.org/.

[47] Sokolova M., Lapalme G. (2009). A systematic analysis of performance measures for classification tasks. Inf. Process. Manag..

[48] Cardoso J.S., Costa J.F. (2007). Learning to classify ordinal data: The data replication method. J. Mach. Learn. Res..

